# Symmetry relations in wurtzite nitrides and oxide nitrides and the curious case of *Pmc*2_1_

**DOI:** 10.1107/S2053273320015971

**Published:** 2021-03-23

**Authors:** Joachim Breternitz, Susan Schorr

**Affiliations:** a Structure and Dynamics of Energy Materials, Helmholtz-Zentrum Berlin für Materialien und Energie, Hahn-Meitner-Platz 1, 14109 Berlin, Germany; b Institute for Chemistry, Universität Potsdam, Karl-Liebknecht-Strasse 24/25, 14476 Potsdam, Germany; c Geosciences, Freie Universität Berlin, Malteserstrasse 74-100, D-12249 Berlin, Germany

**Keywords:** group–subgroup relationships, nitride materials, wurtzite type

## Abstract

Binary III–V nitrides such as AlN, GaN and InN in the wurtzite-type structure have long been considered as potent semiconducting materials because of their optoelectronic properties, amongst others. With rising concerns over the utilization of scarce elements, a replacement of the trivalent cations by others in ternary and multinary nitrides has led to the development of different variants of nitrides and oxide nitrides crystallizing in lower-symmetry variants of wurtzite. This work presents the symmetry relationships between these structural types specific to nitrides and oxide nitrides and updates some prior work on this matter. The non-existence of compounds crystallizing in *Pmc*2_1_, formally the highest subgroup of the wurtzite type fulfilling Pauling’s rules for 1:1:2 stoichiometries, has been puzzling scientists for a while; a rationalization is given, from a crystallographic basis, of why this space group is unlikely to be adopted.

## Abstract 2

Binary and multinary nitrides in a wurtzitic arrangement are very interesting semiconductor materials. The group–subgroup relationship between the different structural types is established.

## 1. Introduction   

GaN and InN in particular are probably some of the most prominent and most important semiconductor materials; indeed, the 2014 physics Nobel Prize was awarded for the invention of blue-emitting GaN LEDs (Nanishi, 2014 ▸). As well as LEDs, other optoelectronic semiconductor devices, such as solar cells, have been realized using alloys of InN and GaN (*e.g.* Aliberti *et al.*, 2010 ▸). However, In, which is needed for bandgap values suitable for visible-light absorption, is a very scarce element, accounting only for 0.16 p.p.m. of the earth’s crust (Webelements, 2020 ▸). While the binary system is limited to trivalent cations to account for the triple negative nitride anion, variations on the cation charges can be realized through more complex substitutions of the cations, such that the overall charge is maintained. In the simplest case, two trivalent cations can, for instance, be replaced by one divalent and one tetravalent cation. This is, for instance, realized in the Zn–IV–V_2_ nitride materials ZnSiN_2_, ZnGeN_2_ and ZnSnN_2_ (Punya *et al.*, 2011 ▸). However, more complex substitutions are also observed in compounds such as Li^(+1)^Al^(+3)^Si_2_^(+4)^N_4_^(−3)^ (Ischenko *et al.*, 2002 ▸) or Zn_3_^(+2)^Mo^(+6)^N_4_^(−3)^ (Arca *et al.*, 2018 ▸) to name just two. The situation can get even more complex when introducing O^2−^ anions, and can lead to complex structure–composition relationships. The crystal structures of many of these materials, however, can be clearly linked to the wurtzite-type structure (Baur & McLarnan, 1982 ▸) and this symmetry relationship can, in turn, be used to rationalize some of the electronic properties of these mater­ials, as the electronic structure is linked to the atomic structure for obvious reasons.

Relationships between crystal structure types are based on the relationships of the underlying symmetries through the use of crystallographic group theory (Müller, 2013 ▸). With the wurtzite type being the aristotype, *i.e.* the crystal structure with the highest symmetry in the system, the lower-symmetry variants, the hettotypes, can be accessed in cascades of group–subgroup descents. While it is beyond the scope of this article to give a conclusive overview of crystallographic group theory, one point is eminently important: when lowering the symmetry from a group to a subgroup, symmetry operations are only lost, and no other symmetry operations are added. This means that a crystal structure in the aristotype can also be expressed in a subgroup, but a crystal structure that genuinely crystallizes in the subgroup type cannot be expressed in the group. The subgroup has higher degrees of freedom which permits shifting of atoms (for instance, out of the centre of tetrahedra), or splitting of crystallographic sites, allowing occupation of different atom types with discrete ordering. One can also make the distinction between subgroups within the same point group, *i.e.* without a change in point-symmetry operations, known as *klassengleiche* subgroups (abbreviated by k), or *isomorphic* subgroups (abbreviated by i) if group and subgroup belong to the same space-group type. These group–subgroup transitions are accompanied by an enlargement of the unit cell or a loss of unit-cell centring. If the translational symmetry is kept and only point-symmetry operations are lost, however, the subgroups are called *translationengleich* (abbreviated by t).^1^ The *Inter­national Tables for Crystallography* Volumes A1 and A (Wondratschek & Müller, 2004 ▸; Hahn, 2005 ▸) are a comprehensive tool for the establishment of relationships between space groups. A wide-ranging discussion of the group–subgroup relationships in wurtzite variants was published some time ago by Baur & McLarnan (1982 ▸), but it does, unfortunately, bear a few in­accuracies at some crucial points. Therefore, we set out to redevelop the symmetry relationships in the system specific to nitrides and oxide nitrides together with barely complete tables of nitrides and oxide nitrides in the different structural types as they appear in the Inorganic Crystal Structure Database (ICSD); we will outline some of the difficulties and pitfalls that can arise in the analysis of the symmetry relations in this system.

## 2. An overview of the wurtzite-related structure types   

**Figure 1:**
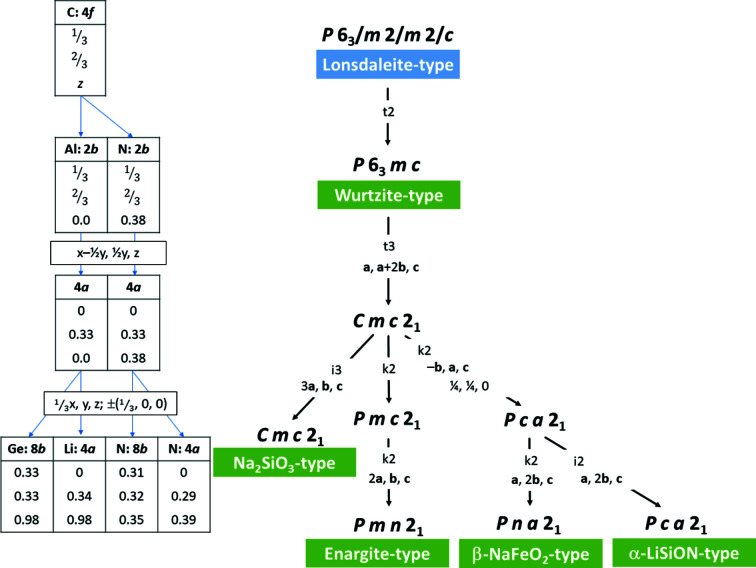
Bärnighausen tree for the wurtzite-derived nitrides and oxide nitrides. The transformation of the basis vector is given below the maximal subgroup-type symbol and index, only if it changes. The respective structure types are given in green. The tables on the left depict the site splitting in the relationship between the Lonsdaleite type and the Na_2_SiO_3_ type. The atomic coordinates for AlN (Schulz & Thiemann, 1977 ▸) and Ge_2_LiN_3_ (Häusler, Niklaus *et al.*, 2018 ▸) are taken from the literature as example cases for the wurtzite type and the Na_2_SiO_3_ type, respectively.

The most powerful tool for the graphical representation of group–subgroup relationships was developed by Bärnig­hausen (1980 ▸), where the relationships are represented in the form of a tree diagram with the highest-symmetry structure standing at the top. Essentially, four subgroups of the wurtzite type are found amongst the wurtzite-derived nitrides and oxide nitrides besides the aristotype (Fig. 1 ▸). For the sake of completeness, Fig. 1 ▸ also contains the relationship between the wurtzite type and the Lonsdaleite type (hexagonal diamond), which can be understood as the prototype for the atom stacking in these materials.

The first complication of this system arises from the transition of the hexagonal wurtzite type in space group *P*6_3_ *mc* to its maximal *translationengleiche* orthorhombic subgroup *Cmc*2_1_. This is because one needs to transition from the hexagonal coordinate system with angles α = β = 90°, γ = 120° to an orthogonal coordinate system with α = β = γ = 90°. This is symbolized as **a**, **a** + 2**b**, **c** in the Bärnighausen tree (Fig. 1 ▸), which relates the basis vectors of the subgroup to those of the group. It also corresponds to a complex transformation of atom coordinates between the two space groups which becomes necessary. Although no structure is observed in the maximal subgroup *Cmc*2_1_, it forms an important link, as it is an intermediate space group to all lower-symmetry variants. If the atomic positions are given in decimals rather than fractions, they are no longer bound by symmetry to specific positions. The second complication arises because all subgroups are in orthorhombic space-group types, where one particular axis setting has been defined as the standard setting based on the symmetry operations. Müller (2013 ▸) advocates for the use of non-standard settings of space groups to avoid unit-cell transformations, but we use the standard settings of the space groups herein to facilitate the use of the work for the wider community. An example of this is the transition from *Cmc*2_1_ to *Pca*2_1_, where *a* and *b* are swapped and *c* is inverted.

While the intermediate space group *Cmc*2_1_ allows for higher degrees of freedom, it still contains only two independent crystallographic sites, one for the anions and one for the cations. To accommodate different cations or anions on distinct crystallographic sites, the symmetry needs to be lowered even further. Lowering the symmetry, the cation and anion sites split in different ways to accommodate different ratios of cations and anions, but also to form different ordering patterns. It has been outlined before that Pauling’s rules are a decisive factor in the way these materials are built (Baur & McLarnan, 1982 ▸; Quayle *et al.*, 2015 ▸). It is interesting to note that the nominally highest-symmetry subgroup for such an octet-rule-obeying arrangement, *Pmc*2_1_, is not found in any existing material.

## 3. The different crystal structure types   

### 3.1. Wurtzite type   

**Figure 2:**
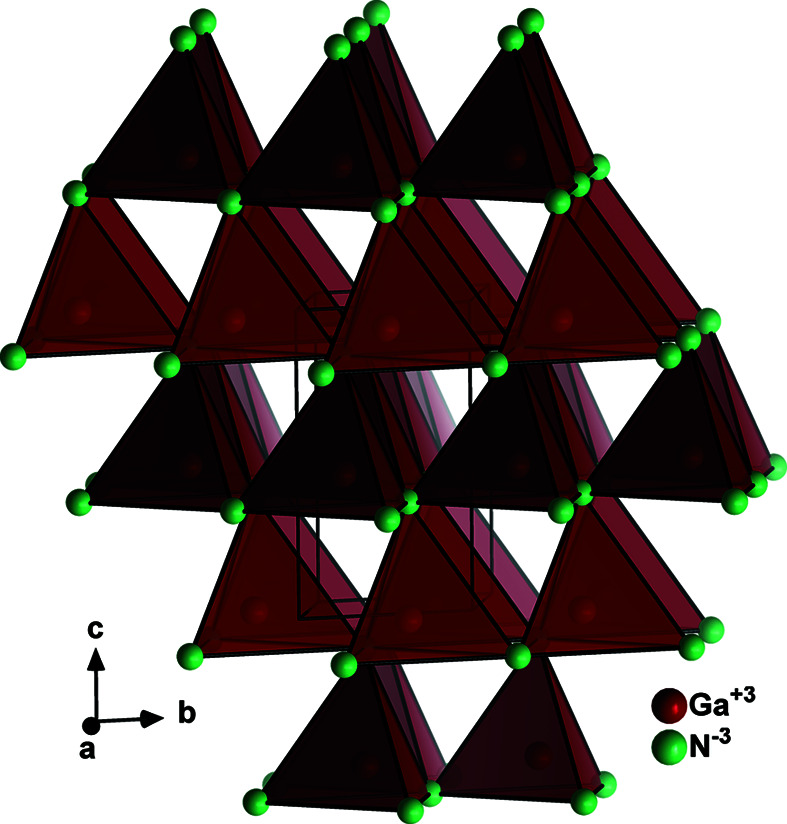
Structure of GaN (Paszkowicz *et al.*, 2004 ▸) in the wurtzite type. All atoms are drawn as generic spheres.

The binary III–V nitrides of main group 3 (apart from BN), namely AlN, GaN, InN and TlN, crystallize in the wurtzite-type crystal structure (Fig. 2 ▸, Table 1 ▸). It is worth noting that the wurtzite-type structure is non-centrosymmetric, *i.e.* it lacks a centre of inversion as symmetry operation. Consequently, all its hettotypes are non-centrosymmetric too. Besides the pure binaries, a vast number of binary alloy compounds exist, where the cations are disordered on the single crystallographic cation site. This feature is used for the effective bandgap tuning in these compounds, for instance in the system Ga_(1−*x*)_In_*x*_N (Jakkala & Kordesch, 2017 ▸). Not only do III–V nitrides form wurtzite-type crystal structures, but nominally ternary systems, such as BeSiN_2_ (Schneider *et al.*, 1979 ▸) and ZnGeN_2_ (Larson *et al.*, 1974 ▸), have been reported to adopt the disordered wurtzite type. In fact, we recently showed that introduction of oxygen on the anion sites leads to the formation of Zn_1+*x*_Ge_1−*x*_N_1−*y*_O_*y*_ compounds in the wurtzite type with disordered cations and anions (Breternitz *et al.*, 2019 ▸). This is in line with multi-cation oxide nitrides that still adopt the wurtzite aristotype, such as Cd_0.25_Zn_1.13_Ge_0.62_ON (Capitán *et al.*, 2000 ▸).

### 3.2. The Na_2_SiO_3_ type   

When the cation site is occupied by more than one atom type, an ordered case is normally energetically and geo­metrically favourable. The latter is easy to quantify through the ionic size of the different cations. If the cations are disordered on one crystallographic site, the difference in coordination environment between the different atom types is only possible within rather strict borders. Therefore, a tendency of the cations to order reduces the strain on the crystal structure, combined with an energetic advantage.

**Figure 3:**
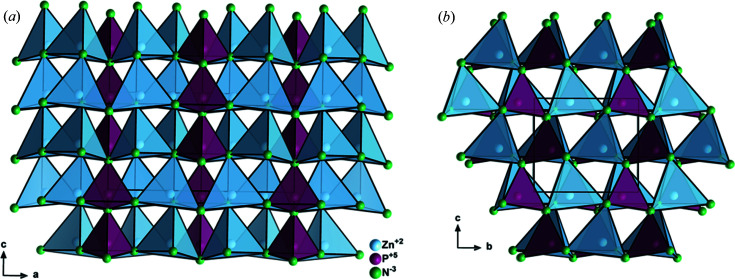
Structure of Zn_2_PN_3_ (Sedlmaier *et al.*, 2011 ▸) in the Na_2_SiO_3_ type with views of the crystallographic *ac* plane (*a*) and the *bc* plane (*b*). Atoms are shown as generic balls.

To allow for an ordering of different cation types on different crystallographic sites, the positions need to be split in agreement with the ratio of the cations in the material. Take Zn_2_PN_3_, for instance (Fig. 3 ▸). The Zn:P ratio is 2:1 and hence a splitting of the crystallographic sites into a different ratio would unavoidably cause disorder on some positions. The simplest 2:1 splitting of the cation 4*a* site in *Cmc*2_1_, as the maximal subgroup of the wurtzite type, is through an *isomorphic* symmetry descent of index three (Fig. 1 ▸). Thereby, the 4*a* site is split into one special 4*a* position with *x* = 0, *i.e.* lying in the *bc* plane, and one 8*b* general position (*x*, *y*, *z*) that imposes no restrictions on the atom positions. Given the multiplicity of the two different sites, there are only half as many atoms on the 4*a* positions as on the 8*b* positions in this arrangement, which are being filled 2:1 in the Na_2_SiO_3_ type (Table 2 ▸). The Zn and P atoms in Zn_2_PN_3_ occupy the Wyckoff positions 8*b* and 4*a*, respectively. It is worth mentioning that the cation site splitting goes along with an anion site splitting. In pure nitrides, these two anion positions are both occupied by nitro­gen, but the site splitting allows for a deviation of the coordination environment for both cation sites.

Taking Zn_2_PN_3_ as an example case for the Na_2_SiO_3_ type (Fig. 3 ▸), it can be easily depicted that the PN_4_ tetrahedra form strands along the *b* directions, which is an effect of the special 4*a* positions on which phospho­rus resides, whereas the ZnN_4_ tetrahedra are interconnecting the strands in three dimensions. It is interesting to note that all nitro­gen atoms are connected to phospho­rus atoms as well as to zinc atoms, with the nitro­gen on 4*a* being connected to two P^5+^ and two Zn^2+^ and the one on 8*b* to one P^5+^ and three Zn^2+^. Therefore, both positions do not strictly fulfil the octet rule as discussed previously, but only approximate Pauling’s rules (George *et al.*, 2020 ▸) with bond strengths of +3.5 and +2.75, respectively.

#### 3.2.1. The ordered defect variant Si_2_N_2_O   

**Figure 4:**
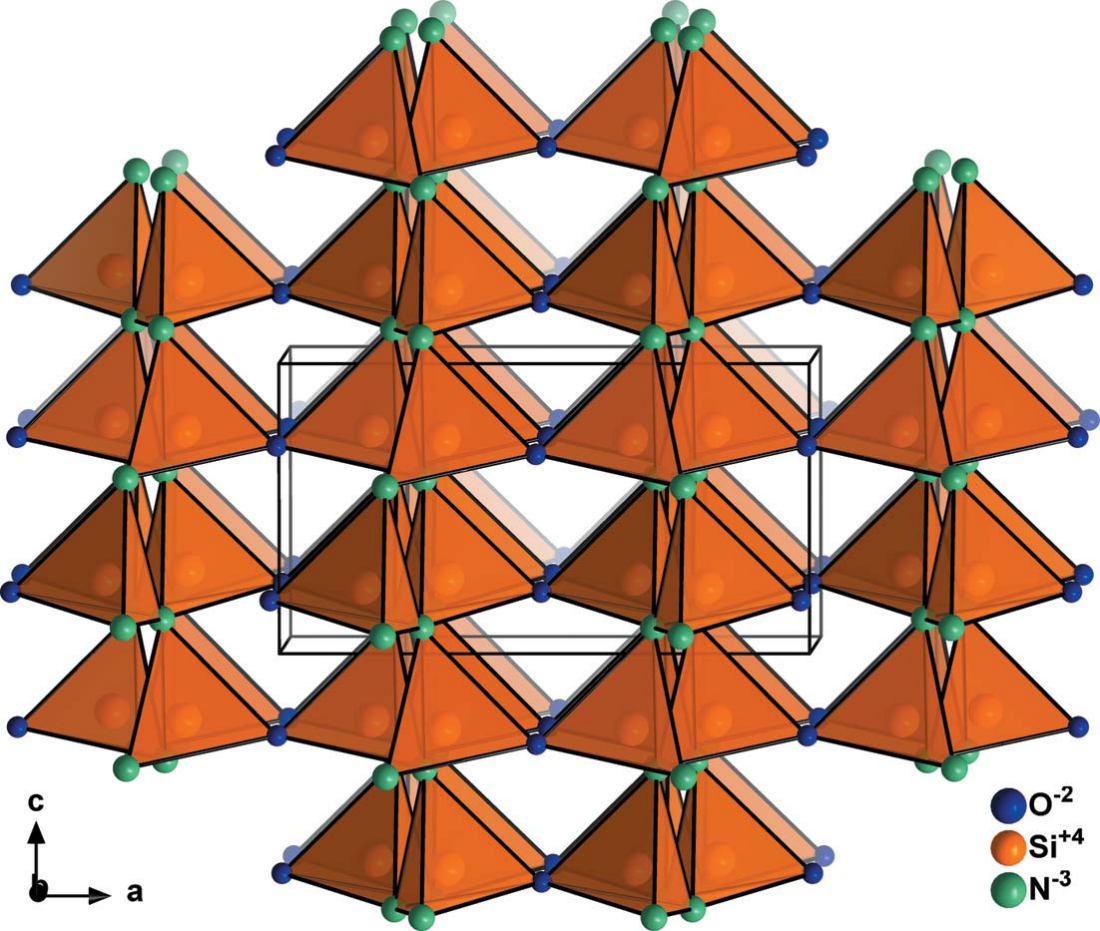
Crystal structure of Si_2_N_2_O (Sjöberg *et al.*, 1991 ▸) shown roughly along the *b* axis. Atoms are drawn as generic balls.

The Si_2_N_2_O type (Fig. 4 ▸ and Table 3 ▸) can be seen as a special case of the Na_2_SiO_3_ type, since both crystallize in the same space group – and would be located at the same position in the Bärnighausen tree – but are different in the occupation of the crystallographic sites. The 4*a* position remains unoccupied, as compared with the Na_2_SiO_3_ type. However, since this class of compounds bears two distinct anions, the anion site splitting mentioned above plays an important role here in that the two crystallographically independent anion sites are occupied by nitro­gen (8*b*) and oxygen (4*a*). In this particular arrangement, every oxygen atom is neighbouring two silicon atoms, whereas every nitro­gen atom is neighbouring three silicon atoms and thereby obeying Pauling’s rules with formal bond strengths of 2 and 3, respectively.

Finally, SiPN_3_, which should be more correctly written as (Si_0.5_P_0.5_)_2_N_3_ since Si and P are sharing sites, can be viewed as an intermediate between the Na_2_SiO_3_ type and the Si_2_N_2_O type, since it only contains one sort of anion, which it shares with the Na_2_SiO_3_ type, but exhibits an unoccupied 4*a* cation position like the Si_2_N_2_O type. In fact, both cations share the general 8*b* position and show no particular order as observed by Baldus *et al.* (1993 ▸). However, this result was determined on the basis of X-ray and electron diffraction only, but P^5+^ and Si^4+^ are isoelectronic ions and would hence scatter in much the same way and therefore not allow a truly reliable determination.

### 3.3. The β-NaFeO_2_ type   

When it comes to a 1:1 ratio of cations, there has been much discussion of the formally highest-symmetry crystal structure that complies with Pauling’s rules in *Pmc*2_1_. This space group is a maximal *klassengleiche* subgroup of *Cmc*2_1_ and is a space group in the transition to the enargite-type structure (see also Figs. 1 ▸ and 6). However, this space group has not been observed and nitrides with a cation ratio of 1:1 are reported to crystallize in the β-NaFeO_2_ type in the space group *Pna*2_1_, which has a unit cell twice as large as that of the hypothetical *Pmc*2_1_ structure. Since this phenomenon has led to some confusion, we will discuss it in more detail in Section 4.

**Figure 5:**
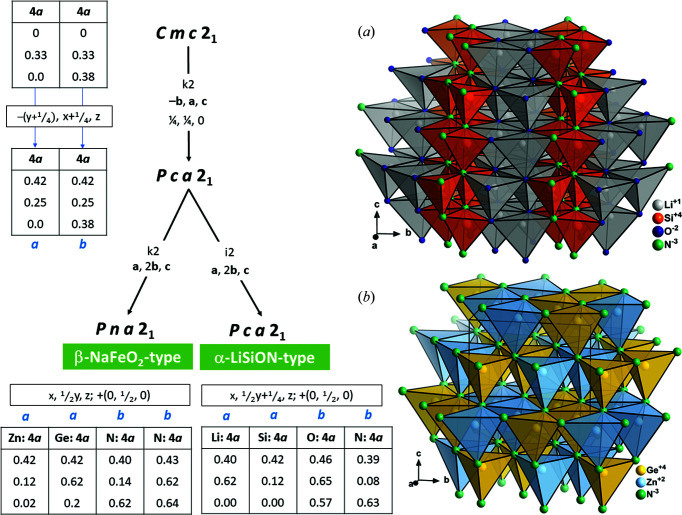
Group–subgroup relationship between the wurtzite subgroup *Cmc*2_1_ and the β-NaFeO_2_-type as well as the α-LiSiON-type structures. Structure representations of the α-LiSiON type (*a*) (Laurent *et al.*, 1981 ▸) and the β-NaFeO_2_ type (*b*) (Häusler, Neudert *et al.*, 2017 ▸) are drawn as general views with atoms as generic spheres. An origin shift (0, 0, −0.5) was applied to the coordinates in the documented structure of LiSiON (Laurent *et al.*, 1981 ▸) to highlight the group–subgroup relationship. We note that the orientation of the polar axis *c* is inverted in the documented structures with respect to the group–subgroup derived one.

One complication that makes the direct comparison of this structure sometimes difficult is that all atoms in the β-NaFeO_2_-type structure lie on general 4*a* positions. Therefore, the choice of the unit-cell origin is arbitrary in this system and may necessitate a shift of the experimentally determined coordinates to reveal the group–subgroup relationship derived ones, as is illustrated in the table in Fig. 5 ▸. This can either be performed manually or through automatic tools, such as the program *COMPSTRU* (de la Flor *et al.*, 2016 ▸). Since this structure obeys Pauling’s rules, every anion is surrounded by two cations of every sort to equalize the charges on every anion position [Fig. 5 ▸(*b*)]. The fact that all atoms lie on general positions further allows a high structural flexibility accommodating cations of distinctly different sizes, for instance (Table 4 ▸).

One particular case of the β-NaFeO_2_ type is the zinc nitride halides. In principle, there are two possible ways to view them in terms of the structure type: one either regards them as a special case, where the two cation types are the same and the anion sites are occupied by different atoms, or as anti-β-NaFeO_2_ type, where cations and anions switch sites. Since all atoms lie on a general position and have the same coordination, both ways lead to the structure and are hence interchangeable from a structural point of view. However, the authors would argue for the latter case as anti-β-NaFeO_2_ type, since one further point needs to be considered: the occupation of the anion positions by two different types with different charges can only obey Pauling’s rule if the cation positions are filled by cations of the same charge, in the simplest case the same cation sort. This mutual dependency is the exact opposite for the pure nitrides in the β-NaFeO_2_ type and the nitride halides should hence be regarded as anti-β-NaFeO_2_ type.

### 3.4. α-LiSiON type   

As the nitride halides demonstrate, the β-NaFeO_2_ type does not allow for an occupation of the cation sites and the anion sites with differently charged ions, while obeying Pauling’s rules at the same time; instead, a different arrangement of the tetrahedra needs to be achieved. The number of next neighbours of the distinct crystallographic sites needs to be different for the different sites. This is achieved through a different symmetry descent from the common intermediate subgroup into the space group *Pca*2_1_ in the α-LiSiON-type structure (Fig. 5 ▸). The cations form planes in the *ac* plane and so do the anions. The effect of this is that every oxygen atom has three lithium and one silicon neighbour, while the nitro­gen atoms have three silicon and one lithium neighbour. While the formal bond strengths of 1.75 and 3.25, respectively, do not perfectly obey Pauling’s rule, they are considerably closer to the expected values than the 2.5 throughout the β-NaFeO_2_ type. Given the rather special arrangement in this class, only two compounds, namely LiSiON (Laurent *et al.*, 1981 ▸) and KGeON (Guyader *et al.*, 1983 ▸), have been experimentally observed in this structure type.

### 3.5. The enargite type   

**Figure 6:**
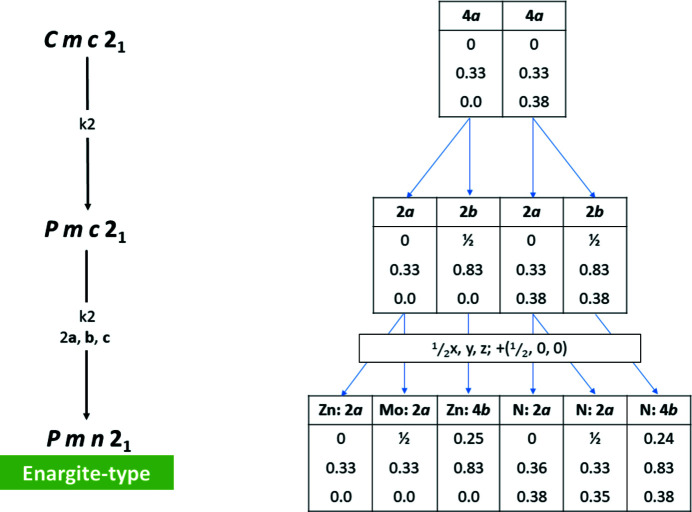
Group–subgroup relationship from the common intermediate subgroup *Cmc*2_1_ into the enargite type. An origin shift (0, 0, 0.73) was applied to the atomic parameters of Zn_3_MoN_4_ (Arca *et al.*, 2018 ▸) to highlight the group–subgroup relationship.

**Figure 7:**
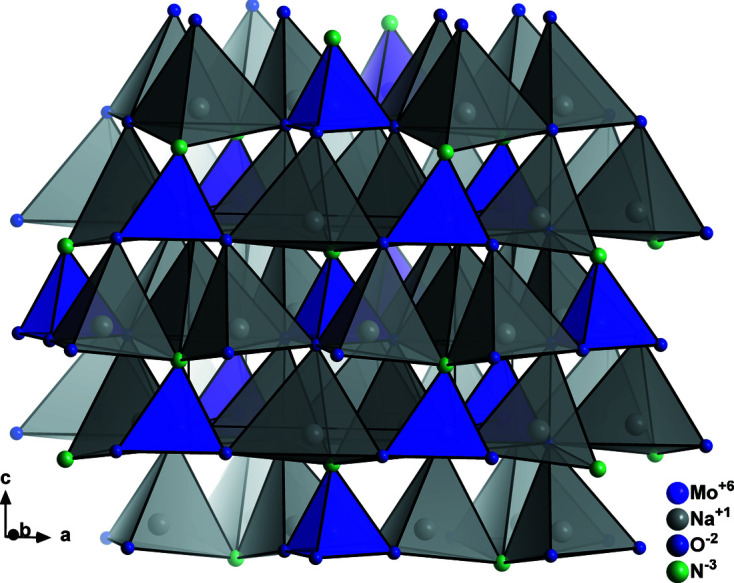
Structure of Na_3_MoO_3_N (Arumugam *et al.*, 2003 ▸) in a general view. Atoms are shown as generic spheres.

Finally, one further structure type is observed in the nitride wurtzite system. Instead of a simple 1:1, or a 2:1 splitting of the crystallographic sites as observed in the β-NaFeO_2_ type and the Na_2_SiO_3_ type, cation and anion positions are split into three crystallographic sites (Fig. 6 ▸) with different multiplicities and thereby allowing a 3:1 occupation of the cations or anions. Taking Na_3_MoO_3_N as an example, sodium occupies a 2*a* and a 4*b* position, while Mo occupies a 2*a* position, with the situation for the anions being analogous. Therefore, the MoN_4_ tetrahedra are isolated within the crystal structure and completely surrounded by NaN_4_ tetrahedra (Fig. 7 ▸). Taking a simple view with Pauling’s rules fails in this structure type, since all anions are surrounded by three sodium cations and one molybdenum cation. This is probably due to the different nature of the Mo—N/O bonding *versus* the Na—N/O bonding, with the former being distinctly more covalent. Therefore, a careful consideration of the bond lengths in addition to the simple counting of nearest neighbours is necessary to rationalize this ordering. This situation is even more obvious in the compound Li_3_SO_3_N, where the S—O/N bonding is clearly covalent, while the Li—O/N bonding is expected to be mostly ionic. Although this structure type shows a considerable degree of complexity, four compounds, Li_3_SO_3_N (Kurzman *et al.*, 2013 ▸), Na_3_MoO_3_N (Arumugam *et al.*, 2003 ▸), Na_3_WO_3_N (Elder *et al.*, 1994 ▸) and Zn_3_MoN_4_ (Arca *et al.*, 2018 ▸), span the range for this class from oxide nitrides to pure nitrides.

## 4. The curious case of *Pmc*2_1_   

In the light of the complexity of group–subgroup relationships and the fact that they are probably rarely taught, problems sometimes occur in the literature that are prone to being replicated in work based on it. They are, however, crucially important for a proper understanding of symmetry relations, and we would like to showcase this for the case of *Pmc*2_1_, a space group that has been postulated for ternary nitrides for a long time (*e.g.* Baur & McLarnan, 1982 ▸; Quayle *et al.*, 2015 ▸; Quayle, 2020 ▸).

As highlighted above, a number of research articles have pointed out that the highest-symmetry subgroup of the wurtz­ite type in which an *AB*N_2_ nitride could crystallize and that obeys Pauling’s rules is not the β-NaFeO_2_ type, but a crystal structure in *Pmc*2_1_. This postulated crystal structure can be found as an intermediate subgroup in the descent to the enargite type (Figs. 1 ▸ and 6 ▸). The difference between the hypothetical crystal structure in *Pmc*2_1_ and the β-NaFeO_2_ type is, indeed, only found in the relative arrangement of the different cations to each other, which led Baur and McLarnan to speculate in 1982 that the energy difference between the two conformations may be small. Quayle *et al.* (2015 ▸) calculated the energy difference between the observed β-NaFeO_2_-type structure and the hypothetical *Pmc*2_1_ structure to be only 13 meV per formula unit in the case of ZnSnN_2_. From this point of view, it is considered interesting why the *Pmc*2_1_ structure has not been observed (and in fact has not been observed for any of the wurtzite series of materials).

**Figure 8:**
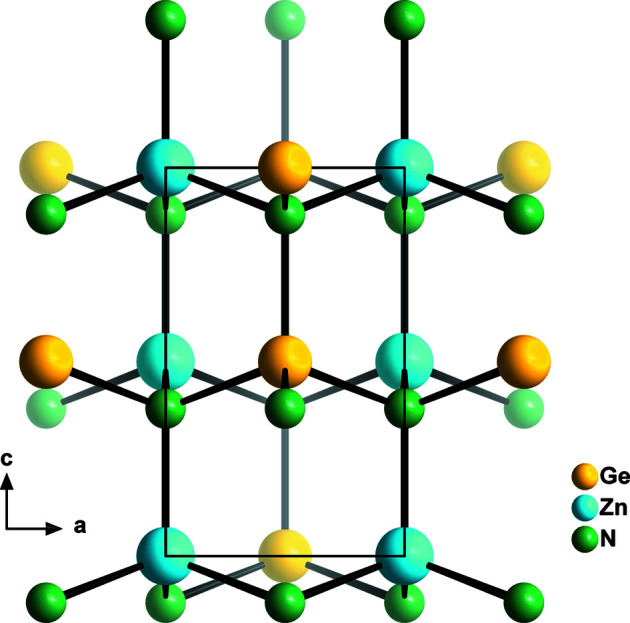
View of ZnGeN_2_ in the hypothetical *Pmc*2_1_ structure along the crystallographic *b* axis. The crystal structure was derived from the wurtzite type using the *TRANSTRU* tool of the Bilbao Crystallographic Server (Aroyo, Perez-Mato *et al.*, 2006 ▸; Aroyo, Kirov *et al.*, 2006 ▸; Aroyo *et al.*, 2011 ▸).

From a crystallographic and crystal chemistry point of view, it is not quite as surprising to find that the structure in *Pmc*2_1_ is not observed. Regarding the group–subgroup relationship as outlined in Figs. 1 ▸ and 6 ▸, it is evident that the crystallographic sites of the cations and anions split from 2*b* Wyckoff sites in the hexagonal wurtzite aristotype into 2*a* and 2*b* sites in *Pmc*2_1_. It is important to remember that Wyckoff sites not only show the multiplicity, but also indicate the site symmetry (which is *m* for both Wyckoff sites). In fact, the 2*a* and 2*b* sites in *Pmc*2_1_ are special positions with the coordinates (0, *y*, *z*) and (½, *y*, *z*), respectively. This essentially means that the two sites can accommodate different atom types, but that they are bound to lie in the *bc* plane (Fig. 8 ▸). This restriction does not only apply to the cations, but is true for the anions too. The restriction to the *bc* plane means that the *M*—N distances (*M*, cations) outside the *bc* plane are critically dependent on the *a*-axis length and this is the same for both types of cations. In essence, the tetrahedral coordination can either strongly distort into a disphenoid,^2^ or will have to remain very similar for both ions. This arrangement cannot be favourable, since a distortion would create a strongly anisotropic bonding situation, which is hardly energetically favourable, and identical tetrahedron sizes for cations of different size would be peculiar, as this is one of the main drivers for ordering. This is in line with the observation of Quayle *et al.* (2015 ▸) that the energy difference between the β-NaFeO_2_-type structure and the hypothetical *Pmc*2_1_ structure is larger for ZnGeN_2_ than for ZnSnN_2_, as the Shannon radii of Zn^2+^ (0.6 Å) and Ge^4+^ (0.39 Å) are more different than those of Zn^2+^ and Sn^4+^ (0.55 Å) (Shannon, 1976 ▸).

One further thing should be considered. It is not the atoms that follow the symmetry of the space group, but the space group that reflects the symmetry of the atomic arrangement. While the space group *Pmc*2_1_ is not a likely choice, as outlined above, the particular arrangement of tetrahedra in this hypothetical structure could well exist. To rectify the symmetry restriction, one needs to lose the mirror planes perpendicular to the *a* axis. This could be done in a *klassen­gleiche* descent of index two into *Pca*2_1_ with a doubling of the *b* axis, or through *translationengleiche* descents into one of the monoclinic space groups *Pc* or *P*2_1_. However, no crystal structure that would correspond to one of these subgroups has been reported in the ICSD.

## 5. Conclusions   

The structural variability of nitrides and oxide nitrides in the wurtzite type and its subgroups is rich and gives rise to many different properties that can be attained in particular arrangements. Combining and comparing these different arrangements and putting them into their group–subgroup relationship can greatly aid the interpretation, but it needs to be performed with great care. In particular, group–subgroup relationships, as they exist in this family, between the hexagonal and the orthorhombic crystal system can be difficult due to the change of basis vectors, as well as those within the orthorhombic crystal system, due to different unit-cell settings. We have developed the relationship between the most important structural types that are found for wurtzite and wurtzite-derived nitrides and explained the differences between them, which mostly reside in the cation arrangements relative to each other. Finally, we showcased why ternary nitrides with a 1:1:2 stoichiometry are unlikely to adopt a structure in the space group *Pmc*2_1_, although much speculation has been devoted to this point. A thorough understanding of the relationship between electronic and atomic structures must, from our perspective, be preceded by a thorough understanding of the atomic structures themselves.

## Figures and Tables

**Table 1:** Some of the nitrides and oxide nitrides in the wurtzite type

Compound	*a*, *c* (Å)	*V* (Å^3^)	Reference
AlN	3.110, 4.980	41.71	Schulz & Thiemann (1977 ▸)
GaN	3.189, 5.186	45.69	Paszkowicz *et al.* (2004 ▸)
InN	3.538, 5.704	61.82	Paszkowicz *et al.* (2003 ▸)
TlN	3.68, 6.01	70.49	Pilyankevich *et al.* (1974 ▸)
BeSiN_2_	2.87, 4.67	33.31	Schneider *et al.* (1979 ▸)
Al_0.49_Ga_0.51_N	3.142, 5.084	43.47	Belousov *et al.* (2010 ▸)
ZnGeN_2_	3.193, 5.187	45.8	Larson *et al.* (1974 ▸)
Ga_0.33_Ge_0.33_Zn_0.33_N	3.192, 5.185	45.74	Suehiro *et al.* (2017 ▸)
Zn_1.23_Ge_0.69_O_0.78_N_1.22_	3.209, 5.193	46.31	Bacher *et al.* (1989 ▸)
Ga_0.87_Zn_0.13_N_0.84_O_0.16_	3.190, 5.184	45.68	Yashima *et al.* (2005 ▸)
Ga_0.91_Zn_0.09_N_0.91_O_0.09_	3.200, 5.199	46.12	Chen *et al.* (2017 ▸)
Al_0.95_Si_0.05_N	3.111, 4.909	41.16	Liu *et al.* (2012 ▸)
Cd_0.25_Ge_0.62_Zn_1.13_N_1.24_O_0.76_	3.200, 5.180	45.93	Capitán *et al.* (2000 ▸)

**Table 2:** Some of the nitrides in the Na_2_SiO_3_ type

Compound	*a*, *b*, *c* (Å)	*V* (Å^3^)	Reference
Si_2_LiN_3_	9.22, 5.30, 4.78	233.45	Orth & Schnick (1999 ▸)
Si_2_(Li_0.99_Eu_0.005_)N_3_	9.21, 5.30, 4.77	233.17	Li *et al.* (2009 ▸)
Na_2_SiN_3_	9.47, 5.50, 4.88	254.11	Jacobs & Mengis (1993 ▸)
Ge_2_LiN_3_	9.56, 5.52, 5.05	266.32	Häusler, Niklaus *et al.* (2018 ▸)
Ge_2_NaN_3_	9.87, 5.78, 5.12	292.25	Guyader *et al.* (1984 ▸)
Mg_2_PN_3_	9.73, 5.64, 4.73	259.62	Schultz-Coulon & Schnick (1997 ▸)
Zn_2_PN_3_	9.38, 5.48, 4.93	252.92	Sedlmaier *et al.* (2011 ▸)
(Al_0.5_Si_0.5_)_2_(Sr_0.5_Ca_0.5_)N_3_	9.83, 5.69, 5.11	285.92	Watanabe & Kijima (2009 ▸)
(Al_0.27_Si_0.69_)_2_CaN_3_	9.76, 5.65, 5.05	278.36	Li *et al.* (2008 ▸)
(Ga_0.5_Si_0.5_)_2_(Ca_0.99_Eu_0.01_)N_3_	9.89, 5.66, 5.08	284.26	Häusler, Neudert *et al.* (2017 ▸)
(Li_0.25_Al_0.25_Si_0.5_)_3_N_3_	9.26, 5.33, 4.87	240.45	Ischenko *et al.* (2002 ▸)
(Al_0.4_Ge_0.6_)_2_(Li_0.2_Ca_0.8_)N_3_	9.98, 5.78, 5.15	296.85	Häusler, Eisenburger *et al.* (2018 ▸)

**Table 3:** Some of the nitrides and oxide nitrides in the Si_2_N_2_O type

Compound	*a*, *b*, *c* (Å)	*V* (Å^3^)	Reference
Si_2_N_2_O	8.87, 5.49, 4.85	236.28	Sjöberg *et al.* (1991 ▸)
Ge_2_N_2_O	9.31, 5.76, 5.11	273.58	Jorgensen *et al.* (1979 ▸)
(Si_0.72_Al_0.28_)_2_(N_0.72_O_0.28_)_2_O	8.93, 5.50, 4.86	238.43	Lindqvist *et al.* (1991 ▸)
(Si_0.5_P_0.5_)_2_N_3_	9.02, 5.28, 4.70	223.63	Baldus *et al.* (1993 ▸)

**Table 4:** Some of the nitrides, oxide nitrides and nitride halides in the (anti-)β-NaFeO_2_ type

Compound	*a*, *b*, *c* (Å)	*V* (Å^3^)	Reference
BeSiN_2_	4.98, 5.75, 4.67	133.67	Eckerlin (1967 ▸)
MgSiN_2_	5.27, 6.47, 4.99	170.18	Bruls *et al.* (2000 ▸)
MgGeN_2_	5.50, 6.63, 5.18	188.94	Häusler, Niklaus *et al.* (2018 ▸)
MnSiN_2_	5.27, 6.52, 5.07	174.26	Esmaeilzadeh *et al.* (2006 ▸)
MnGeN_2_	5.50, 6.66, 5.25	192.35	Häusler, Niklaus *et al.* (2018 ▸)
ZnSiN_2_	5.25, 6.28, 5.02	165.47	Häusler, Schimmel *et al.* (2017 ▸)
ZnGeN_2_	5.47, 6.45, 5.19	182.92	Häusler, Schimmel *et al.* (2017 ▸)
Zn(Si_0.5_Ge_0.5_)N_2_	5.38, 6.33, 5.11	174.02	Endo *et al.* (1992 ▸)
(Zn_0.99_Mn_0.01_)GeN_2_	5.46, 6.44, 5.18	182.23	Zhang *et al.* (2010 ▸)
Cd_0.8_Ge_0.87_(N_0.55_O_0.45_)_2_	5.77, 6.81, 5.40	211.83	Capitán *et al.* (2000 ▸)
Zn_2_NCl	6.12, 7.39, 5.94	268.6	Liu *et al.* (2013 ▸)
Zn_2_NBr	6.21, 7.65, 6.09	289.46	Liu *et al.* (2013 ▸)
Zn_2_N(Br_0.53_Cl_0.47_)	6.17, 7.54, 6.03	280.17	Li *et al.* (2016 ▸)

## Footnotes

1 There is a degree of overlap in the nomenclature between subgroup types t, k, i and indices (Müller, 2013 ▸; p. 135) and the corresponding symmetry reductions (*e.g.* Müller, 2013 ▸; p. 134). Since both are so closely related to each other, we argue that a term like t3 could be synonymously used for a *translationengleiche* subgroup of index three as well as a *translationengleiche* symmetry descent of index three.

2 We note that, in the orthorhombic crystal system, the tetrahedra are all strictly speaking distorted into disphenoids, since they are no longer bound by symmetry to maintain equilateral triangle faces. As the distortions are generally small in these compounds, and in order to allow a direct comparison of the higher-symmetry structures with the lower-symmetry ones, we use the term tetrahedron throughout this article, whether it is a regular or irregular polyhedron.
